# Risk Factors and Burden of Occupational Injuries Among Railway Wagon Repair Workers in Madhya Pradesh: A Cross-Sectional Study

**DOI:** 10.7759/cureus.90385

**Published:** 2025-08-18

**Authors:** Shubhanshu Gupta, Mansi Gupta, Anshul Gupta

**Affiliations:** 1 Community Medicine, Government Medical College, Datia, IND; 2 Microbiology, Bundelkhand Medical College, Sagar, IND; 3 Orthopedics, Bundelkhand Medical College, Sagar, IND

**Keywords:** injury severity, occupational injury, ppe use, railway workers, workplace safety

## Abstract

Background

Occupational injuries continue to pose a significant public health challenge worldwide, particularly in low- and middle-income countries such as India, where industrial safety measures frequently fail to keep pace with rapid infrastructural development. Railway wagon repair workers constitute a vulnerable population due to their exposure to mechanical, ergonomic, and environmental hazards.

Objective

The objective of the study is to assess the prevalence and severity of occupational injuries and identify associated sociodemographic and occupational factors among railway wagon repair workers in Datia, Madhya Pradesh.

Methods

A cross-sectional study was conducted from July 2023 to June 2024 among 327 railway wagon repair workers who had sustained work-related injuries. Data were collected using a structured questionnaire and analyzed with Jamovi software. Injury severity was assessed using a custom 10-point scale. Multivariate linear and binary logistic regression models were employed to identify predictors of injury severity and injury occurrence, respectively.

Results

The prevalence of occupational injuries was 59.0% (N = 193). Male workers, older age groups, and unskilled workers reported a higher incidence of injury; however, these associations were not statistically significant. Only the type of occupation significantly predicted injury severity, with unskilled workers experiencing more severe injuries (β = 1.165; p = 0.008). No variable significantly predicted injury occurrence. Model fit for both severity (adjusted R² = 0.0134) and occurrence (McFadden’s R² = 0.0178) was poor, suggesting that the identified factors explain only a small portion of the variability and that severity and occurrence are likely influenced by multiple unmeasured factors. These findings should, therefore, be interpreted with caution regarding their predictive value.

Conclusion

Unskilled workers face a higher risk of sustaining severe occupational injuries in railway wagon repair settings. Although no single factor independently predicted injury occurrence, the findings highlight the need for improved occupational safety practices, particularly through targeted skill development, strict enforcement of personal protective equipment (PPE) use, and customized safety training programs. Longitudinal studies using validated injury assessment tools are recommended to further clarify causal pathways.

## Introduction

Workplace injuries remain a significant public health issue worldwide, especially in low- and middle-income countries where industrial growth outpaces the development and enforcement of occupational health and safety (OHS) regulations. According to the 2016 Global Burden of Disease Study [1], occupational exposures accounted for approximately 1.53 million deaths and 76.1 million disability-adjusted life years (DALYs). Most of these deaths were attributable to injury-related hazards, particulate matter, carcinogens, and ergonomic stressors. Men and older adults bore the greatest burden, primarily due to gender roles and longer durations of labor-intensive employment [1].

In India, transport- and industry-related injuries constitute a significant portion of the overall injury burden, with the working-age population being the most affected. Between 1990 and 2019, the number of road traffic injuries in India increased by 38%, leading to reduced labor productivity and placing an increasing strain on the healthcare system. Train-related injuries have also emerged as a major contributor to trauma-related morbidity and mortality [2]. A multicenter study reported that most victims of train-related trauma were young men (mean age: 33 years), with a median Injury Severity Score (ISS) of 9, a median hospital stay of 11 days, and a hospital mortality rate of 17.4%. The most common causes of death were severe head injuries and cardiac arrest in the emergency department [3].

There remains a gap in epidemiological data on occupational injuries among railway workers, particularly those engaged in physically demanding and hazardous tasks such as wagon repair. Existing literature has predominantly focused on railway passengers and unauthorized workers. Industry data from West Bengal indicate that nearly 28% of workers experience occupational injuries annually, including sharp tool cuts, fractures, burns, and head trauma. These injuries are associated with inadequate technical training, high-risk job profiles, insufficient use of safety equipment, and low awareness of safety protocols [4]. Similar findings have been reported in Ethiopia, where construction workers lacking access to personal protective equipment (PPE), adequate safety training, and job satisfaction are more prone to workplace injuries [5]. Beyond physical hazards, behavioral and environmental factors also significantly contribute to injury development. Human behaviors such as inattention, insufficient safety monitoring, and poorly designed warning systems have been identified as contributing factors to workplace accidents. An analytic network process (ANP)-decision-making trial and evaluation laboratory (DEMATEL) model study identified 19 high-risk behavioral triggers, ranging from cognitive biases to inadequate equipment maintenance [6]. Ergonomic stressors, including prolonged standing, poor posture, and repetitive movements, have been shown to cause musculoskeletal injuries, particularly in service occupations in urban India [7].

Although research from various occupations highlights occupational health risks, studies specifically on railway wagon repair are scarce. Existing work is mostly descriptive and lacks an analytical assessment of risk factors. This study was conducted to fill that gap by assessing the prevalence and severity of occupational injuries and by identifying sociodemographic and work-related risk factors among railway wagon repair workers in Datia district, Madhya Pradesh. The site was chosen because of its large workforce in this sector and the limited research available on their occupational health.

## Materials and methods

Study design and setting

A facility-based, cross-sectional study was conducted at the railway wagon repair workshop located in the urban area of Datia district, Madhya Pradesh, India. The workshop environment is a critical setting for investigating occupational accidents, as it is characterized by physically demanding tasks, confined spaces, and exposure to hazardous machinery.

Study duration and population

The study was conducted over a one-year period, from July 2023 to June 2024. Workers employed at the workshop who sustained workplace-related injuries during this time were enrolled and assessed.

Sample size estimation

The sample size was calculated using the formula for cross-sectional studies, i.e.,

$N=Z^{2}pq/L^{2}$

where Z = 1.96 for a 95% confidence level, p = 25.7% (estimated prevalence from previous literature [8]), q = 100% − p = 74.3%, and an absolute precision (L) of 5%. The resulting sample size was approximately 294. To account for an expected dropout rate of 10%, the final target sample size was increased to 327 participants.

Inclusion and exclusion criteria

Workers aged 18 years and older who were currently employed at the railway wagon repair workshop and had sustained a work-related injury during the study period were included in the study. Workers who had been injured outside the workplace, those with pre-existing chronic conditions (e.g., cardiovascular, musculoskeletal, psychiatric, or neurological disorders), and pregnant women were excluded from the study.

Data collection instrument

Data were collected using a structured, self-developed questionnaire (Appendix) created by the research team to address the study’s specific objectives, with its content rigorously reviewed by subject experts to ensure face and content validity. A non-validated, study-specific tool was chosen due to the absence of a standardized instrument covering the unique contextual and operational aspects under investigation, allowing inclusion of locally relevant items and terminology for greater applicability to the research setting. It captured information on sociodemographic characteristics, occupational profile, shift and seasonal factors, use of PPE, safety training, causes and types of injury, and injury history.

Injury severity was assessed using a self-developed 10-point scale, specifically designed for field-based evaluation of workplace injuries in resource-constrained settings. This scale was developed in preference to established trauma scoring systems (e.g., Abbreviated Injury Scale (AIS), ISS, New ISS (NISS)), which have limited applicability for rapid, on-site assessments in occupational field studies and are not intended for predicting clinical outcomes. Scores ranged from 1 (least severe) to 10 (most severe) and incorporated factors such as injury type (e.g., laceration, contusion, and fracture), number of affected anatomical sites, need for medical attention, and number of sick days.

Ethical considerations

The study protocol was approved by the Institutional Ethics Committee for Biomedical and Human Participants Research (137/CM/GMC/IECBMHR/2023) dated April 15, 2023. Written informed consent was obtained from all participants prior to data collection. Participant anonymity, confidentiality, and voluntary participation were strictly maintained throughout the study.

Statistical analysis

Data were entered into Microsoft Excel (Microsoft Corp., Redmond, WA) and analyzed using Jamovi statistical software, version 2.3.28 (Jamovi Project, Sydney, Australia). The Chi-squared test assessed associations between categorical variables such as demographics, risk behaviors, and injury categories. Multivariate linear regression analyzed continuous outcomes (ISS), reporting adjusted beta coefficients with 95% confidence intervals (CIs), while binomial logistic regression evaluated binary outcomes (presence or absence of specific injury types), providing adjusted odds ratios (ORs).

## Results

Prevalence of occupational injuries and associated sociodemographic factors

A total of 327 industrial workers participated in the study, with an occupational injury prevalence of 59.0% (n = 193). Injury occurrence was higher among male workers (54.4%) compared to female workers; however, this difference was not statistically significant (p = 0.366). Other factors, including age group, education level, PPE use, safety training, type of occupation, and work shift duration, showed no significant association with injury prevalence (Table 1).

**Table 1:** Association Between Sociodemographic and Occupational Variables With Reported Workplace Injuries Among Railway Wagon Repair Workers (N = 327) Data presented as frequency (n) and percentage (%), calculated out of total participants (N = 327). Statistical analysis was performed using Pearson’s Chi-squared (χ²) test. A p-value of <0.05 was considered statistically significant NS: not significant

Variable	Category	Injured, N (% out of total)	Not injured, N (% out of total)	Total, N (% out of total)	χ²	p-value
Sex	Female	15 (4.6%)	7 (2.1%)	22 (6.7%)	0.818	0.366^NS^
	Male	178 (54.4%)	127 (38.8%)	305 (93.3%)		
Education level	Illiterate	31 (9.5%)	31 (9.5%)	62 (19.0%)	3.19	0.526^NS^
	Primary	37 (11.3%)	27 (8.3%)	64 (19.6%)		
	Middle	41 (12.5%)	26 (8.0%)	67 (20.5%)		
	High School	42 (12.8%)	27 (8.3%)	69 (21.1%)		
	Graduate+	42 (12.8%)	23 (7.0%)	65 (19.9%)		
PPE used	Yes	144 (44.0%)	98 (30.0%)	242 (74.0%)	0.0897	0.765^NS^
	No	49 (15.0%)	36 (11.0%)	85 (26.0%)		
Safety training	Yes	98 (30.0%)	66 (20.2%)	164 (50.2%)	0.0734	0.786^NS^
	No	95 (29.0%)	68 (20.8%)	163 (49.8%)		
Occupation type	Unskilled	61 (18.7%)	39 (11.9%)	100 (30.6%)	0.239	0.887^NS^
	Semi-skilled	66 (20.2%)	48 (14.7%)	114 (34.9%)		
	Skilled	66 (20.2%)	47 (14.4%)	113 (34.6%)		
Age group	18–27	41 (12.5%)	25 (7.6%)	66 (20.2%)	1.67	0.797^NS^
	28–37	41 (12.5%)	23 (7.0%)	64 (19.6%)		
	38–47	30 (9.2%)	25 (7.6%)	55 (16.8%)		
	48–57	36 (11.0%)	26 (8.0%)	62 (19.0%)		
	58+	45 (13.8%)	35 (10.7%)	80 (24.5%)		
Shift hours	8	114 (34.9%)	76 (23.2%)	190 (58.1%)	0.205	0.903^NS^
	12	59 (18.0%)	44 (13.5%)	103 (31.5%)		
	24	20 (6.1%)	14 (4.3%)	34 (10.4%)		

Predictors of injury severity

A multivariable linear regression model was used to identify predictors of injury severity. The ISS was significantly higher among unskilled workers compared to semi-skilled workers (β = 1.165, standard error (SE) = 0.435, p = 0.008). Other variables, including sex, age, PPE use, safety training, and shift hours, were not significant predictors of injury severity. The model explained only 1.3% of the variance in injury severity, indicating a poor fit (adjusted R² = 0.0134). Furthermore, the assumptions of normality and homoscedasticity were violated (Shapiro-Wilk p < 0.001; Breusch-Pagan p = 0.021) (Table 2).

**Table 2:** Multivariable Linear Regression Analysis of Predictors of Injury Severity Score Among Workers (N = 327) Data are presented as unstandardized regression coefficients (β), 95% confidence intervals (CIs), t-statistic (t), standard error (SE), and p-values. The model assesses the association between occupational and demographic variables and the total injury severity score p-value < 0.05 was considered statistically significant **p < 0.01; ***p < 0.001; NS: not significant Reference categories: female (sex), 18–27 years (age), no personal protective equipment (PPE) use, no safety training, semi-skilled (occupation), 8-hour shift (work shift) Dependent variable: self-rated Injury Severity Score (continuous, 1–10 scale)

Predictor variable	Estimate (β)	SE	t	p-value	95% CI
Intercept	4.713	1.317	3.58	<0.001***	(2.121, 7.304)
Sex (male vs. female)	0.839	0.704	1.19	0.234^NS^	(−0.547, 2.226)
Age group (≥58 vs. 18–27)	0.975	0.521	1.87	0.062^NS^	(−0.051, 2.001)
PPE use (yes vs. no)	−0.113	0.406	−0.28	0.781^NS^	(−0.912, 0.685)
Safety training (yes vs. no)	−0.138	0.346	−0.40	0.69^NS^	(−0.819, 0.543)
Occupation (unskilled vs. semi-skilled)	1.165	0.435	2.68	0.008**	(0.310, 2.020)
Work shift (12 vs. 8 hours)	−0.342	0.379	−0.90	0.367^NS^	(−1.088, 0.403)

Predictors of injury occurrence

In the binary logistic regression analysis, no variable independently predicted the likelihood of experiencing an occupational injury. Although illiterate workers had lower odds of injury compared to graduates (OR = 0.51; 95% CI: 0.24-1.07; p = 0.076), this association was not statistically significant. The model demonstrated a poor fit (Akaike information criterion (AIC) = 473; McFadden's R² = 0.0178) (Table 3).

**Table 3:** Binomial Logistic Regression with 95% CIs Predicting Occurrence of Occupational Injury (N = 327) Data are presented as OR, 95% confidence intervals (CIs), Z-score (Z), standard error (SE), and p-values. The model evaluates the association between potential risk factors and the likelihood of sustaining a severe injury p-value < 0.05 was considered statistically significant The model's goodness-of-fit was evaluated using deviance = 435, Akaike information criterion (AIC) = 473, and McFadden’s R² = 0.0178, indicating limited explanatory power *p < 0.05; NS: not significant

Predictor	β estimate	SE	Z	p-value	Odds ratio (OR)	95% CI for OR
Intercept	1.291	0.646	1.996	0.046*	3.64	1.02-12.92
Age group (Ref: 18–27)						
28–37	0.066	0.374	0.178	0.859^NS^	1.07	0.51-2.23
38–47	–0.303	0.387	–0.783	0.434^NS^	0.74	0.34-1.58
48–57	–0.101	0.37	–0.273	0.785^NS^	0.9	0.44-1.87
58+	–0.244	0.351	–0.695	0.487^NS^	0.78	0.39-1.56
Sex (male vs. female)	–0.450	0.489	–0.919	0.358^NS^	0.64	0.24-1.66
Education level (Ref: graduate+)						
High School	–0.249	0.37	–0.675	0.5^NS^	0.78	0.38-1.61
Illiterate	–0.670	0.377	–1.777	0.076^NS^	0.51	0.24-1.07
Middle	–0.279	0.38	–0.734	0.463^NS^	0.76	0.36-1.59
Primary	–0.314	0.371	–0.845	0.398^NS^	0.73	0.35-1.51
Occupation (Ref: semi-skilled)						
Skilled	–0.006	0.282	–0.020	0.984^NS^	0.99	0.57-1.73
Unskilled	0.124	0.291	0.425	0.671^NS^	1.13	0.64-2.00
Work shift (Ref: 8 hours)						
12 hours	–0.105	0.256	–0.408	0.683^NS^	0.9	0.54-1.49
24 hours	0.041	0.393	0.103	0.918^NS^	1.04	0.48-2.25
Safety training (yes vs. no)	0.101	0.234	0.429	0.668^NS^	1.11	0.70-1.75
Cause of injury (Ref: caught between)						
Fall of object	–0.190	0.371	–0.511	0.61^NS^	0.83	0.40-1.71
Machine	–0.288	0.369	–0.780	0.435^NS^	0.75	0.36-1.55
Poor safety environment	–0.332	0.347	–0.956	0.339^NS^	0.72	0.36-1.42
Struck against	0.043	0.347	0.125	0.901^NS^	1.04	0.53-2.06

## Discussion

This study evaluated the prevalence and causes of work-related injuries among railway wagon repair workers in urban North India. We found that 25.4% of these workers had sustained injuries, indicating a significant occupational health concern in this hazardous profession. These findings align with previous research, such as a study conducted in Pakistan, which reported varying injury rates across different occupations, underscoring specific workplace risks [9]. Our results revealed that male workers, older workers (aged 58 years and above), unskilled workers, and those lacking safety training or PPE were more likely to experience injuries. Among these factors, being unskilled was the only statistically significant predictor of injury compared to semi-skilled workers (β = 1.165, p = 0.008). This supports other studies emphasizing the critical role of job function and skill level in injury risk, particularly in physically demanding occupations like construction [10]. Older age was associated with a higher risk of injury in our study, consistent with research indicating that older workers tend to have fewer but more severe injuries due to reduced physical resilience and slower recovery [11]. Male workers in our study experienced more injuries, consistent with findings suggesting that men face greater physical risks and may underreport injuries compared to female workers [12]. This contrasts with healthcare settings, where women, especially nurses, report more injuries from sharp objects [13]. A review of mine workers identified that the lack of ergonomic training and failure to use PPE increased injury risk [14]. In our study, PPE use was associated with a reduction in injury risk (β = -0.113), highlighting the importance of PPE in industrial repair work. Additionally, work environment and shift length are major contributors to workplace injury risk. Although workers on 12-hour shifts did not exhibit higher odds of injury, longer shifts are known to increase fatigue and injury risk, as demonstrated in other studies on construction workers [15]. Furthermore, a study found that each 1°C increase in temperature raised injury risk by 1%, particularly during heatwaves in humid regions, which is relevant to the location of our study [16].

Noman et al. emphasized the need for industry- and occupation-specific analyses and recommended customized OHS strategies [9]. Our study supports this view, demonstrating that railway wagon repair workers face unique risks, including mechanical, ergonomic, and environmental hazards that require targeted interventions. The various causes of injury, such as being struck by objects, slipping, and mechanical trauma, indicate that injuries result from multiple factors. Therefore, policies must address a broad range of issues. Dodoo and Al-Samarraie also showed that injury patterns vary by region, underscoring the importance of adapting safety regulations to local contexts [10].

Limitations

Firstly, the cross-sectional design of this study limits the ability to infer causal relationships between risk factors and injuries. Secondly, reliance on self-reported data may have introduced recall bias and social desirability bias, potentially leading to the underestimation of the true injury prevalence. Thirdly, the ISS employed was not based on validated trauma scoring systems, which may limit the external comparability of the results. Additionally, the study focused exclusively on workers who reported injuries, excluding those without injuries, thereby introducing potential selection bias. Furthermore, objective measurements of ergonomic strain and environmental stressors, such as heat exposure, were not undertaken. Consequently, the findings may have limited generalizability beyond similar railway repair settings in central India.

## Conclusions

This study found that occupational injuries are common among railway wagon repair workers, with unskilled workers experiencing more severe injuries than others. Although factors such as age, use of PPE, and length of work shifts were assessed, none were found to significantly predict the likelihood of injuries. This suggests that workplace injuries result from a combination of factors, and the statistical models used in this study could explain only part of the risk. Focused safety measures such as improving worker skills, ensuring regular and proper use of PPE, and providing training tailored to specific job tasks could help lower injury severity in high-risk groups. Future research using long-term follow-up and reliable injury measurement tools is needed to better understand the causes and design more effective prevention strategies.

## Appendices

**Table 4:** Self-Developed Structured Questionnaire for Sociodemographic, Occupational, Injury, and Safety Perception Assessment Among Rail Wagon Workshop Workers This structured questionnaire was self-developed by the investigators and has not undergone formal psychometric validation. It was specifically designed to collect data across four key domains: sociodemographic details, occupational characteristics, history and severity of occupational injuries, and workers’ perceptions regarding safety practices at the workplace. The tool included closed-ended and multiple-response items and was administered through face-to-face interviews. Injury severity was assessed using a self-developed 10-point scale designed specifically for contextual, field-based evaluation of workplace injuries. This scale ranged from 1 (indicating the least severity) to 10 (indicating the highest severity) and incorporated multiple parameters, including the type of injury (e.g., laceration, contusion, and fracture), the number of affected anatomical sites, the requirement for medical attention, and the number of workdays lost due to injury. It is important to emphasize that this scale was not derived from established trauma scoring systems such as the Abbreviated Injury Scale (AIS), Injury Severity Score (ISS), or New Injury Severity Score (NISS), nor was it intended for clinical outcome prediction

Section A: Sociodemographic Information	Response
1. Age (in completed years):	____________
2. Gender:	☐ Male ☐ Female ☐ Other
3. Education level:	☐ No formal education ☐ Primary ☐ Secondary ☐ Higher Secondary ☐ Graduate & above
4. Marital status:	☐ Married ☐ Unmarried ☐ Widowed/separated
Section B: Occupational Profile	
5. Job role:	☐ Skilled ☐ Semi-skilled ☐ Unskilled ☐ Supervisor
6. Years of experience in this workshop:	____________
7. Average daily shift duration:	☐ ≤8 hours ☐ 9–12 hours ☐ >12 hours
8. Safety training received:	☐ Yes ☐ No
9. Availability of personal protective equipment (PPE):	☐ Yes ☐ No
10. PPE used during work (tick all that apply):	☐ Gloves ☐ Helmet ☐ Goggles ☐ Safety shoes ☐ Apron ☐ Ear protection
Section C: Injury Profile (If "No" in Q11, skip to Section D)	
11. Have you sustained any injury while working here?	☐ Yes ☐ No
12. Month/season of injury:	☐ Summer ☐ Monsoon ☐ Winter
13. What was the mechanism of injury?	☐ Machine-related ☐ Tool-related ☐ Fall/slip ☐ Chemical exposure ☐ Electrocution ☐ Other: ____________
14. Type of injury sustained (tick all that apply):	☐ Laceration ☐ Abrasion ☐ Contusion ☐ Fracture ☐ Burn ☐ Eye injury ☐ Amputation
15. Part of body injured:	☐ Hand ☐ Fingers ☐ Legs ☐ Thigh ☐ Face/eyes ☐ Back ☐ Multiple
16. Injury severity score (1 = mild to 10 = severe):	____________
17. Was medical attention required?	☐ Yes ☐ No
18. Number of days absent from work due to injury:	____________
Section D: Perception and Workplace Safety	
19. In your opinion, what was the primary reason for the injury?	☐ Poor machine condition ☐ Negligence ☐ Lack of training ☐ No PPE ☐ Crowded workspace ☐ Other: ____________
20. Is the workplace environment safe overall?	☐ Yes ☐ No ☐ Can’t say
